# An Efficient Acoustic Density Estimation Method with Human Detectors Applied to Gibbons in Cambodia

**DOI:** 10.1371/journal.pone.0155066

**Published:** 2016-05-19

**Authors:** Darren Kidney, Benjamin M. Rawson, David L. Borchers, Ben C. Stevenson, Tiago A. Marques, Len Thomas

**Affiliations:** 1 Centre for Research into Ecological and Environmental Modelling, University of St Andrews, St Andrews, United Kingdom; 2 IUCN SSC Primate Specialist Group, Hanoi, Vietnam; 3 Centro de Estatística e Aplicações da Universidade de Lisboa, Faculdade de Ciências da Universidade de Lisboa, Lisboa, Portugal; University of South Carolina, UNITED STATES

## Abstract

Some animal species are hard to see but easy to hear. Standard visual methods for estimating population density for such species are often ineffective or inefficient, but methods based on passive acoustics show more promise. We develop spatially explicit capture-recapture (SECR) methods for territorial vocalising species, in which humans act as an acoustic detector array. We use SECR and estimated bearing data from a single-occasion acoustic survey of a gibbon population in northeastern Cambodia to estimate the density of calling groups. The properties of the estimator are assessed using a simulation study, in which a variety of survey designs are also investigated. We then present a new form of the SECR likelihood for multi-occasion data which accounts for the stochastic availability of animals. In the context of gibbon surveys this allows model-based estimation of the proportion of groups that produce territorial vocalisations on a given day, thereby enabling the density of groups, instead of the density of calling groups, to be estimated. We illustrate the performance of this new estimator by simulation. We show that it is possible to estimate density reliably from human acoustic detections of visually cryptic species using SECR methods. For gibbon surveys we also show that incorporating observers’ estimates of bearings to detected groups substantially improves estimator performance. Using the new form of the SECR likelihood we demonstrate that estimates of availability, in addition to population density and detection function parameters, can be obtained from multi-occasion data, and that the detection function parameters are not confounded with the availability parameter. This acoustic SECR method provides a means of obtaining reliable density estimates for territorial vocalising species. It is also efficient in terms of data requirements since since it only requires routine survey data. We anticipate that the low-tech field requirements will make this method an attractive option in many situations where populations can be surveyed acoustically by humans.

## Introduction

Spatially explicit capture-recapture (SECR) methods are becoming increasingly popular for estimating population densities of cryptic species. The main advantage of these techniques is their ability to implicitly estimate the effective sampling area of a capture-recapture experiment by taking account of the spatial information contained in the locations of the detectors [1–3]. Unlike conventional capture-recapture techniques, SECR methods are therefore able to provide direct estimates not only of abundance, but also population density. Furthermore, by modelling the relationship between detection probability and the distance between animal and detector, SECR methods are able to model heterogeneity in capture probabilities due to animal location, which causes biased inference if left unmodelled [2].

Standard SECR models have been used to estimate population density for a variety of species, including horned lizards from visual trapping studies [3], minke whales using bottom-mounted hydrophones [4] and tigers from camera traps arrays [5, 6]. [7] also used an extended SECR model to estimate bird density from recordings of their vocalisations and supplementary data on the location of each detection in the form of received signal strength. They showed that use of this supplementary data can improve inference. [8] generalised this approach and showed that the incorporation of various types of additional information on animal location can improve inference from SECR methods. This generalised framework has since been applied to estimate frog abundance using recapture data and signal time-of-arrival data from a fixed microphone array [9].

Population surveys of gibbons (Family Hylobatidae) are an ideal example of the potential benefit of using an SECR approach. The majority of gibbon species are either Endangered or Critically Endangered [10] and accurate estimates of population density are therefore vital to the success of conservation efforts [11]. Distance sampling, a popular tool for visual surveys of wildlife [12], is generally inappropriate for gibbons since visual detections tend to be rare, due to low densities and cryptic behaviour, and therefore a considerable amount of survey effort is needed in order to yield sufficiently large sample sizes, particularly for the most threatened species. As gibbon species form fairly stable family groups and make loud territorial calls that can be heard over large distances [11], most previous attempts to estimate the density of gibbon populations have been based on acoustic surveys (e.g. [13–15]).

The design of acoustic surveys for gibbons has traditionally followed guidance provided by [13]. A common approach uses replicate arrays of 3–4 listening posts positioned in suitable habitat, from which calling groups are detected and where the posts in each array are close enough to allow the groups to be detected from multiple posts. The time of the calls and the estimated bearings to the calling groups are recorded and the estimated locations of detected groups are mapped via triangulation using the estimated bearings. Density estimates are then obtained by dividing the number of detected groups by an estimate of the area covered by the survey. Gibbon surveys must also account for the fact that groups do not vocalise every day; indeed calling frequency may change between groups, species, seasons and weather conditions (e.g. [14, 16–18]). To derive estimates of group density therefore, either the calling probability must be estimated and the density of calling groups adjusted accordingly, or surveys must be carried out over multiple days until all groups present in the area are assumed to have been detected.

A major difficulty with this approach lies in estimating the size of the covered area (see [11] for a detailed discussion of this topic). Previous surveys have typically estimated the covered area for each array by defining a circular listening zone around each listening post and then calculating the union of these zones. One way of determining the radius of the listening zones is to use an estimate of the maximum distance over which gibbon calls can be heard, with a common choice being 1500m [19]. However, this procedure is likely to be prone to error; underestimation of the maximum listening distance for example, will result in an underestimate of the covered area and an overestimate of density. If this uncertainty is not taken into account, then standard error estimates will be negatively biased and the precision of density estimates will be overestimated. Furthermore, use of a maximum hearing distance in this way implicitly assumes that detection probability is equal to one for all distances less than or equal to the listening zone radius, which may be an unrealistic assumption.

A second approach to choosing the listening zone radius is to use a pre-determined distance within which all groups are assumed to have been detected. Density is then estimated using the number of detected groups whose locations have been mapped within the delineated zone. However, there are three main drawbacks with this approach. Firstly, discarding the information contained in the detections mapped outside the listening zone will decrease the precision of the density estimate. Secondly, it is sensitive to errors associated with the bearing estimates, which translate into uncertainty in terms of the mapped locations. This may result in groups within the zone being excluded and groups outside being included, two processes that cannot be assumed to cancel out. Thirdly, in practice there is still likely to be imperfect detection within the delineated zone, which will introduce an additional source of bias.

Recognising the issue of imperfect detection, [15] employed an alternative technique to estimate the listening radius, applying distance sampling methods to estimated distances to detected groups to obtain an estimate of the *effective* radius [12]. This represents an improvement on previous methods, since it relaxes the unrealistic assumptions regarding the relationship between detection probability and distance by allowing the detection function parameters to be estimated from the survey data. However, this approach still has some significant drawbacks. Firstly, no consideration is given to the effect of error in the distance estimation process, which in the case of acoustic surveys may be large. Secondly, the approach uses an overly simplistic concept of the *effective sampling area*. This can be thought of as being equivalent to the covered area from a survey with a step detection function (i.e. where detection probability is equal to 1 within a certain radius and 0 beyond) in which the expected number of detected groups is the same as that from the actual survey. The approach used by [15] is valid for isolated posts, but not for arrays of multiple, overlapping posts. The effective sampling area for the latter case is more appropriately derived by using the estimated detection function to construct a *detection surface* over the listening post array—which gives the probability of a calling group at a given location being detected by *at least one* listening post—and calculating the volume contained by the surface.

SECR provides a natural alternative for analysing data of this type and addresses many of the disadvantages of traditional estimation methods. Importantly, by accounting for imperfect detection through the use of a detection function and using the spatial information contained in the capture histories, it dispenses with the need for precise mapping of group locations and explicit delineation of the covered area. The ability to incorporate supplementary information on detection also allows error in the bearing estimation process to be accounted for via the inclusion of a bearing error model. In addition, being likelihood based, it produces reliable, model-based estimates of uncertainty for all model parameters and allows objective model selection criteria, such as AIC [20], to be used for model selection.

Here we apply an SECR model using estimated bearings in addition to spatial capture data to a set of single-occasion acoustic survey data from a population of gibbons in order to estimate calling group density. We then conduct a simulation study to evaluate the performance of the estimator. In the interest of informing future monitoring schemes we investigate a set of alternative listening post array designs by simulation.

We then present an extension of the SECR likelihood for multi-occasion data that deals with the fact that gibbon groups do not vocalise every day. We model this situation in general terms as a stochastic process in which animals are available for detection on each sampling occasion with probability *ρ* (which must be estimated from the data) using a binary random effect to represent availability. The modification provides an integrated, model-based solution for estimating the effective sampling area and daily calling probability simultaneously. It therefore allows the density of groups to be estimated, which is a more meaningful quantity than the density of calling groups. It requires recapture data across a minimum of two occasions—something that is achievable for gibbons by comparing the inferred locations of detected groups across consecutive survey days (via bi-angulation or triangulation of the estimated bearings) [13, 14]. We illustrate the new method via a simulation study and discuss its potential applications.

## Materials and Methods

All fieldwork associated with this research was approved and conducted in cooperation with the Forestry Administration of the Royal Government of Cambodia, the relevant government agency who controls and owns the permanent forest estate in which the survey was conducted. No animals ethics approvals were required as the research did not require contact with the study animals. No animals were sampled or collected during this work and no experimental manipulation applied.

### SECR likelihood using estimated bearings

We use a special case of the general SECR likelihood framework presented by [8] which includes supplementary bearings data. Generic notation is used throughout this section. In the case of gibbon surveys however, the term ‘animal’ can be replaced with ‘calling group’, and ‘detector’ can be replaced with ‘listening post’.

A general form for the SECR likelihood, including supplementary bearings data and assuming a uniform distribution of animals, can be written as follows,

$$\begin{array}{c} L\left(\phi ,\theta ,\gamma \right)=Po\left(n;\lambda \left(\phi ,\theta \right)\right)\int_{R^{2}}f_{X\Omega Y}\left(X,\Omega ,Y|n;\phi ,\theta ,\gamma \right)\;dX, \end{array}$$ (1)

where *ϕ*, ***θ*** and *γ* are model parameters representing the unknown animal density, detection function parameters and bearing error parameters respectively, *n* is the number of detected animals, ***X*** is their (unobserved) locations, **Ω** is their capture histories and ***Y*** is the estimated bearing to each detection. The likelihood is composed of two parts: (i) a Poisson sub-model for the number of detections, and (ii) a probability density of locations, capture histories and estimated bearings given the sample size, integrated over all possible animal locations. We introduce these in turn.

In the Poisson sub-model, the observed sample size, *n*, is a Poisson random variable with expectation,

$$\begin{array}{c} \lambda \left(\phi ,\theta \right)=\int_{R^{2}}\phi p.\left(x;\theta \right)dx=\phi \int_{R^{2}}p.\left(x;\theta \right)dx, \end{array}$$ (2)

where *p*.(***x***; ***θ***) is the probability that an animal at location ***x*** is detected by at least one detector. The function *p*.(***x***; ***θ***) is often referred to as the ‘detection surface’.

The specific formulation of the detection surface depends on the type of detectors being used. Listening posts represent an example of *proximity detectors* in SECR terminology which, unlike physical traps, are able to detect animals without detaining them and therefore enable recapture data to be obtained from a single survey occasion. For proximity detectors, assuming independence of detections across detectors (conditional on animal location), the detection surface can be expressed as follows,

$$\begin{array}{c} p.\left(x;\theta \right)=1-\prod_{k=1}^{K}\left[1-p_{k}\left(x;\theta \right)\right], \end{array}$$ (3)

where *p*_*k*_(***x***; ***θ***) is the detection function, giving the probability of detection at detector *k* as a function of animal location ***x*** and the parameter vector ***θ***. In this analysis we consider two common choices of detection function,

$$\begin{array}{cc} \text{the }half\;normal: & p_{k}\left(x;\theta \right)=\theta_{0}\text{ exp}\left(-\frac{d_{k}{\left(x\right)}^{2}}{2\theta_{1}^{2}}\right), \end{array}$$ (4)

$$\begin{array}{cc} \text{and the }hazard\;rate: & p_{k}\left(x;\theta \right)=\theta_{0}\left\{1-\text{exp}\left[-{\left(\frac{d_{k}\left(x\right)}{\theta_{1}}\right)}^{-\theta_{2}}\right]\right\}, \end{array}$$ (5)

where the distance between the animal and detector *k* is represented as a function, *d*_*k*_, of animal location ***x***, and where the parameters *θ*_0_, *θ*_1_ and *θ*_2_ determine the intercept, scale and shape of the detection function respectively.

The second sub-model in the likelihood in Eq (1) is defined in terms of the joint density of locations, capture histories and estimated bearings of detected animals. Since the animal locations are unobserved, they are integrated out of the model—in other words, the locations are treated as random effects. The integrand in this sub-model can be defined as a product of three components:

$$\begin{array}{c} f_{X\Omega Y}\left(X,\Omega ,Y|n;\phi ,\theta ,\gamma \right)=\prod_{i=1}^{n}f_{X}\left(x_{i};\phi ,\theta \right)P\left(\omega_{i}|x_{i};\theta \right)f_{Y}\left(y_{i}|\omega_{i},x_{i};\gamma \right) \end{array}$$ (6)

where *f*_*X*_ (***x***_*i*_; *ϕ*, ***θ***) is the probability density function (pdf) of the location of detected animal *i*, *P*(***ω***_*i*_ ∣ ***x***_*i*_; ***θ***) is the probability mass function (pmf) of the capture history data for animal *i*, and *f*_*Y*_ (***y***_*i*_ ∣ ***ω***_*i*_, ***x***_*i*_; *γ*) is the pdf of the estimated bearings for animal *i*.

The pdf for the location of animal *i* can be defined as a scaled form of the detection surface, such that the volume is equal to 1,

$$\begin{array}{c} f_{X}\left(x_{i};\phi ,\theta \right)=\frac{\phi p.(x_{i};\theta )}{\lambda (\phi ,\theta )}. \end{array}$$ (7)

An expression for *P*(***ω***_*i*_ ∣ ***x***_*i*_; ***θ***), the probability of the capture history for animal *i*, conditional on detection, is obtained through an application of Bayes’ theorem,

$$\begin{array}{c} P\left(\omega_{i}|x_{i};\theta \right)=\frac{\prod_{k=1}^{K}Bern\left(\omega_{ik},p_{k}\left(x_{i};\theta \right)\right)}{p.(x_{i};\theta )}, \end{array}$$ (8)

where the unconditional probability for *ω*_*ik*_, the binary indicator for the capture history of animal *i* at detector *k*, is modelled using a Bernoulli distribution with parameter *p*_*k*_(***x***_*i*_; ***θ***).

Finally, the sub-model for the bearing data can be expressed as a circular pdf, *f*_*Y*_ (*y*_*ik*_; *γ*), which gives the probability density of the estimated bearing for animal *i* at detector *k* as a function of the parameter vector *γ*,

$$\begin{array}{c} f_{Y}\left(y_{i}|\omega_{i},x_{i};\gamma \right)=\prod_{k=1}^{K}{\left[f_{Y}\left(y_{ik};\gamma \right)\right]}^{\omega_{ik}}, \end{array}$$ (9)

where the probability density for the bearing data for animal *i* at detector *k* is 1 if the capture history, *ω*_*ik*_, is zero (i.e. if the animal was not detected). We consider two options for the circular distribution,

$$\begin{array}{cc} \text{the }von\;Mises: & f_{Y}\left(y;\gamma \right)=\frac{\text{exp}(\gamma \text{ cos}\left(y-b_{k}\left(x\right)\right)}{2\pi I_{0}\left(\gamma \right)}, \end{array}$$ (10)

$$\begin{array}{cc} \text{and the }wrapped\;Cauchy: & f_{Y}\left(y;\gamma \right)=\frac{1}{2\pi }\frac{\text{sinh}\left(\gamma \right)}{\text{cosh}\left(\gamma \right)-\text{cos}\left(y-b_{k}\left(x\right)\right)}, \end{array}$$ (11)

where the expected (i.e. average) bearing estimate between the animal in question and detector *k* is represented as a function, *b*_*k*_, of animal location ***x*** and scale parameter *γ* (and where *I*_0_ is the modified Bessel function of order zero).

Given a set of data on capture histories of detected animals (**Ω**) and estimated bearings to each detection (***Y***), parameter estimates for density (*ϕ*), detection function (***θ***) and bearing error (*γ*) can be obtained by maximising the log of the likelihood in Eq (1). In practice the required integrations in Eqs (1) and (2) are typically evaluated numerically using a grid of points (see [21] for an example of this approach).

### Case study: Estimating gibbon population density

Acoustic SECR data and bearing data were collected by surveyors at listening posts on a population of northern yellow-cheeked gibbon *Nomascus annamensis* [22], in the Veun Sai-Siem Pang Conservation Area in northeastern Cambodia between 1st Feb and 30th March 2010. Like all gibbons, northern yellow-cheeked gibbon are strongly territorial with mated pair and offspring (2–5 animals per group on average) defending their territories against other groups, with little overlap. Territory size has not been extensively studied in the taxon, however based on closely related gibbon species it is expected to be upwards of 30 ha. Previous studies have estimated that yellow-cheeked gibbons can be heard from a maximum of of 1.5km in conditions similar to the survey region [23–25].

**Fig 1:**
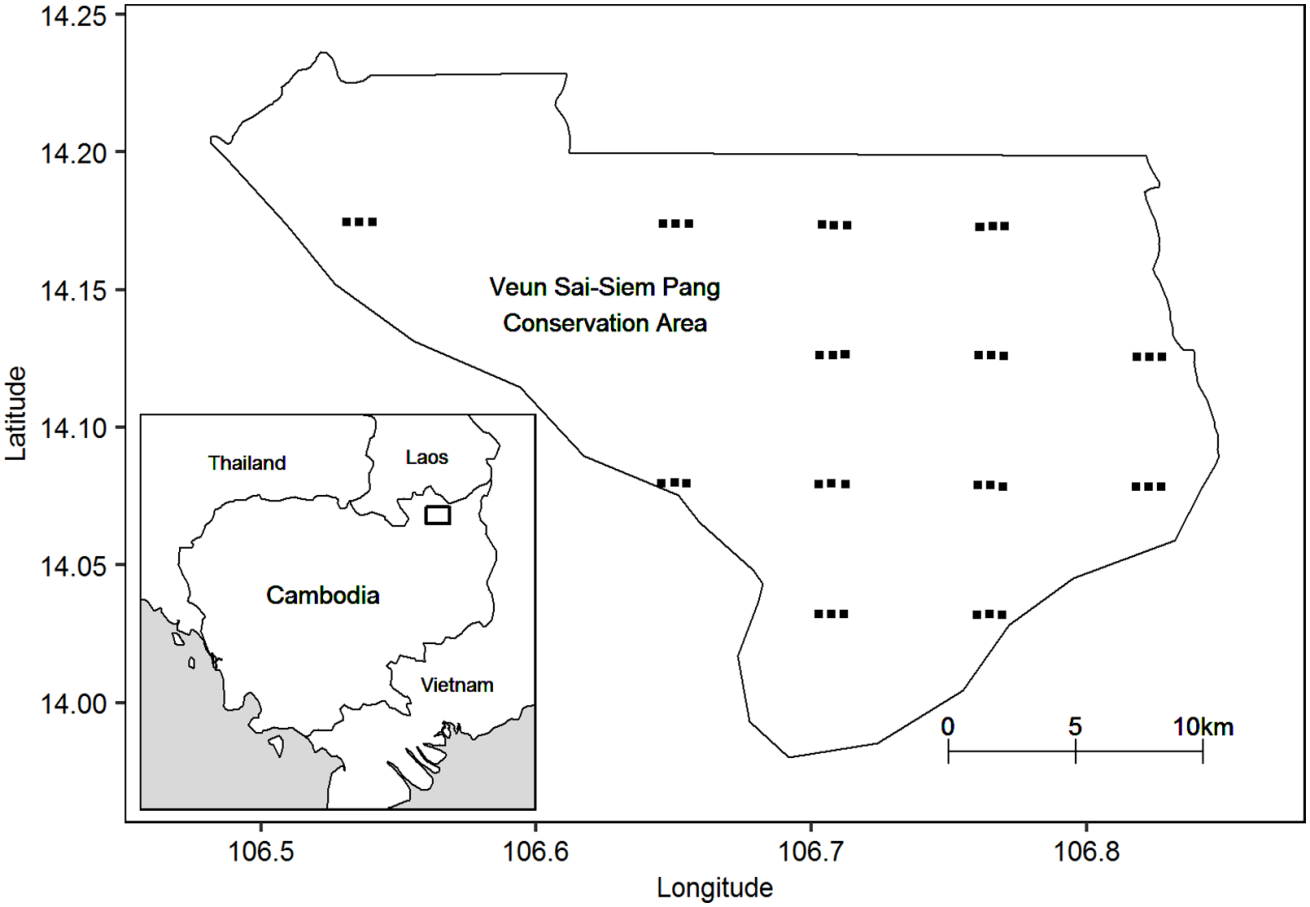
Listening post locations. Each of the 13 detector arrays for the case study survey consisted of a linear arrangement of three listening posts spaced 500m apart.

A total of 13 replicate survey locations were sampled, which were spaced at least 4km apart in order to avoid groups being detected at more than one survey location. Each survey location consisted of a 1 by 3 linear array of listening posts spaced 500m apart to allow calls to be detected at more than one listening post within an array (see Fig 1 and S1 Appendix for listening post locations). Each location was surveyed on a single day during the survey period, with data being collected during a 4-hour observation period between 5.30am and 9:30am. Ten observers participated in the survey, each of whom underwent four days of training in gibbon survey methods at the site prior to the start of the survey (even though many had significant existing experience). Observers at each listening post recorded the timing of calls and an estimated compass bearing to each detected group. Recaptures—i.e. detections for the same group at more than one listening post—were determined *post hoc* by the field team using the estimated bearings and detection times. Where bearings from detections at multiple posts crossed, and start and finish times of vocalisations were similar, these detections were assumed to represent the same group.

Detections of solo males were ignored for the purposes of the analysis since it is difficult to determine whether they represent roaming individuals or members of a group [14]. Following their removal, the survey data consisted of 123 separate detections of 77 calling groups, 36 of which were detected at more than one listening post.

Four candidate SECR models were constructed with different combinations of the detection function and bearing error sub-models described above. All models used the following assumptions: (i) detections were made independently; (ii) the shape of the detection function was the same for all listening posts; (iii) calling groups at zero distance from a listening post were detected with certainty (i.e. *θ*_0_ = 1 in Eqs (4) and (5)); (iii) bearing estimation was unbiased for all listening posts (i.e. the expected bearing *b*(***x***) was equal to the true bearing); and (iv) the precision of bearing estimates was the same at all listening posts.

All models were fitted using the statistical software environment R [26]. A function to evaluate the marginal SECR log-likelihood, using a numerical method to approximate the integration step, was optimised using the nlm function (which implements a Newton-type algorithm) to obtain maximum likelihood parameter estimates. Data from each array was treated as independent and the full likelihood was therefore represented as a product of Eq (1). Confidence intervals for parameter estimates for the preferred model were derived using a parametric bootstrap with 999 re-samples. (See S1 Appendix for further details of the model fitting and model selection procedures.)

### Simulation 1: Comparing survey designs

A simulation study was carried out to assess the performance of the gibbon density estimator with a variety of survey designs. Performance was assessed in terms of the bias and variance of the estimator.

Separate simulations were conducted for each of the following listening post arrangements: (i) 1 by 3 linear (as used in the original survey); (ii) equilateral triangle; (iii) 1 by 4 linear; and (iv) 2 by 2 square. Each arrangement was assessed separately for three different listening post spacing distances: 500m (as used in the original survey), 750m and 1000m.

Each simulation was performed by carrying out 5000 iterations of the following steps, using the fitted parameters of the preferred model from the case study analysis as the underlying truth: (i) generate true locations for an artificial population within a sufficiently large buffer zone around the listening posts (i.e. large enough to contain all plausible locations for the detected groups); (ii) generate a single-occasion capture history and a corresponding set of bearing estimates from the population; (iii) fit the preferred model to these data to obtain parameter estimates, using the true parameter values to initialise the fitting procedure and the same integration grid as in the case study analysis.

To investigate the utility of the estimated bearing data, the simulations were also repeated a second time in which the simulated data were analysed using standard SECR estimation—i.e. using the capture history data only and ignoring the bearing data.

### SECR likelihood with stochastic availability

We extend the SECR likelihood to accommodate stochastic availability of animals for detection on each sampling occasion. The likelihood is generalisable to any situation in which recaptures are available across occasions and is applicable to SECR methods in general, with or without supplementary data on location. We again use notation in generic form, but note that in the case of gibbons the probability of being available for detection can be interpreted as the probability of calling.

We introduce the partially unobserved random vector ***α***, which is a vector of indicator variables *α*_*is*_ that take the value 1 if animal *i* was available for detection on occasion *s* and 0 otherwise. If animal *i* is detected on occasion *s* then *α*_*is*_ must be equal to 1 and its value is therefore observed. However, if animal *i* is not detected on occasion *s* then two possible events could have occurred—either animal *i* was not available, or animal *i* was available but was not detected—in which case the value of *α*_*is*_ is unknown. We also introduce the parameter *ρ* which represents the probability that a given animal is available on a given occasion, i.e. *P*(*α*_*is*_ = 1) = *ρ*. As a consequence of this modification, the density parameter *ϕ* is reinterpreted as the density of animals, instead of the density of available animals. Note also that by having a single parameter we implicitly assume that the probability of being available is constant across animals and occasions.

To construct the extended form of the SECR likelihood we first redefine the detection function as giving the probability of an animal being detected on occasion *s*, given it’s location ***x*** *and given that the animal is available for detection on that occasion*, i.e. *p*_*ks*_(***x***; ***θ*** ∣ *α*_*is*_ = 1).

Next we obtain the following expression for the detection surface by summing over ***α***, which is defined as a Bernoulli random effect with parameter *ρ* (see S2 Appendix for derivation),

$$\begin{array}{c} p.\left(x_{i};\theta ,\rho \right)=1-\prod_{s=1}^{S}\left\{\left(1-\rho \right)+\rho \prod_{k=1}^{K}\left[1-p_{ks}\left(x_{i};\theta |\alpha_{is}=1\right)\right]\right\}. \end{array}$$ (12)

Details on the derivation of Eq (12) are provided in S2 Appendix. Using this expression we obtain a modified form for *λ*, the Poisson rate parameter,

$$\begin{array}{c} \lambda \left(\phi ,\theta ,\rho \right)=\phi \int_{R^{2}}p.\left(x;\theta ,\rho \right)dx. \end{array}$$ (13)

The conditional probability of the capture history data also needs to be reformulated by summing over the random vector ***α***. This leads to the following expression (see S2 Appendix for derivation),

$$\begin{array}{c} P\left(\omega_{i}|x_{i};\theta ,\rho \right)=\frac{\prod_{s=1}^{S}\left(1-\rho \right)\left(1-\omega_{i.s}\right)+\rho \prod_{k=1}^{K}Bern\left(\omega_{iks},p_{ks}\left(x_{i};\theta |\alpha_{is}=1\right)\right)}{p.(x_{i};\theta ,\rho )}, \end{array}$$ (14)

where *ω*_*i*.*s*_ is an indicator that takes values 1 if animal *i* was heard by at least one detector on occasion *s* and 0 otherwise.

Note that the likelihood presented in the previous section is a special case of this likelihood, since if the probability of being available is known in advance to be equal to 1, then Eqs (12), (13) and (14) simplify to the multi-occasion versions of Eqs (3), (2) and (8). Since the estimation of *ρ* is not dependent on the presence of supplementary information on the locations, this modification is also applicable to standard SECR models.

### Simulation 2: Stochastic availability

To investigate the performance of the estimator with stochastic availability a second simulation study was performed. Since previous research has suggested that the proportion of gibbons groups in a population that call on a given day is in the region of 50% (e.g. [15, 18, 27]) a value of 0.5 was used as the true value of the availability parameter *ρ*.

The stochastic availability model likelihood was fitted to 5000 simulated datasets using a similar procedure to the first simulation study. In this case a single survey design was used: a 1 by 4 linear array of listening posts with 1000m spacing, a design which performed well in the first simulation study. Because the calling probability was 0.5, the true density of groups was chosen to be double the density of calling groups used in the first simulation in order to generate the same mean sample size (given that the availability parameter was set to 0.5). The half normal detection function with the same scale parameter as the first simulation study was used, but in this case the intercept (*θ*_0_) was set at 0.75 and estimated in each iteration, in order to demonstrate the general applicability of the likelihood and the absence of identifiability issues between *θ*_0_ and *ρ*. The same bearing model and integration grid was used as in the first simulation study and the true parameter values were used to initialise the fitting procedure.

A three-occasion capture history was generated from each simulated dataset. This was achieved by first simulating a three-occasion capture history from the population assuming that all groups in the population called on each occasion. Then to simulate stochastic availability, each occasion-specific capture history for each group (i.e. each ***ω***_*is*_) was set to zero for all listening posts with probability 1 − *ρ*. The positions of gibbon groups were also kept constant between survey occasions. Whilst this is unlikely to be a realistic assumption in practice, it enabled bias and variance properties of the parameter estimates to be more easily determined.

## Results

### Case study analysis

**Fig 2:**
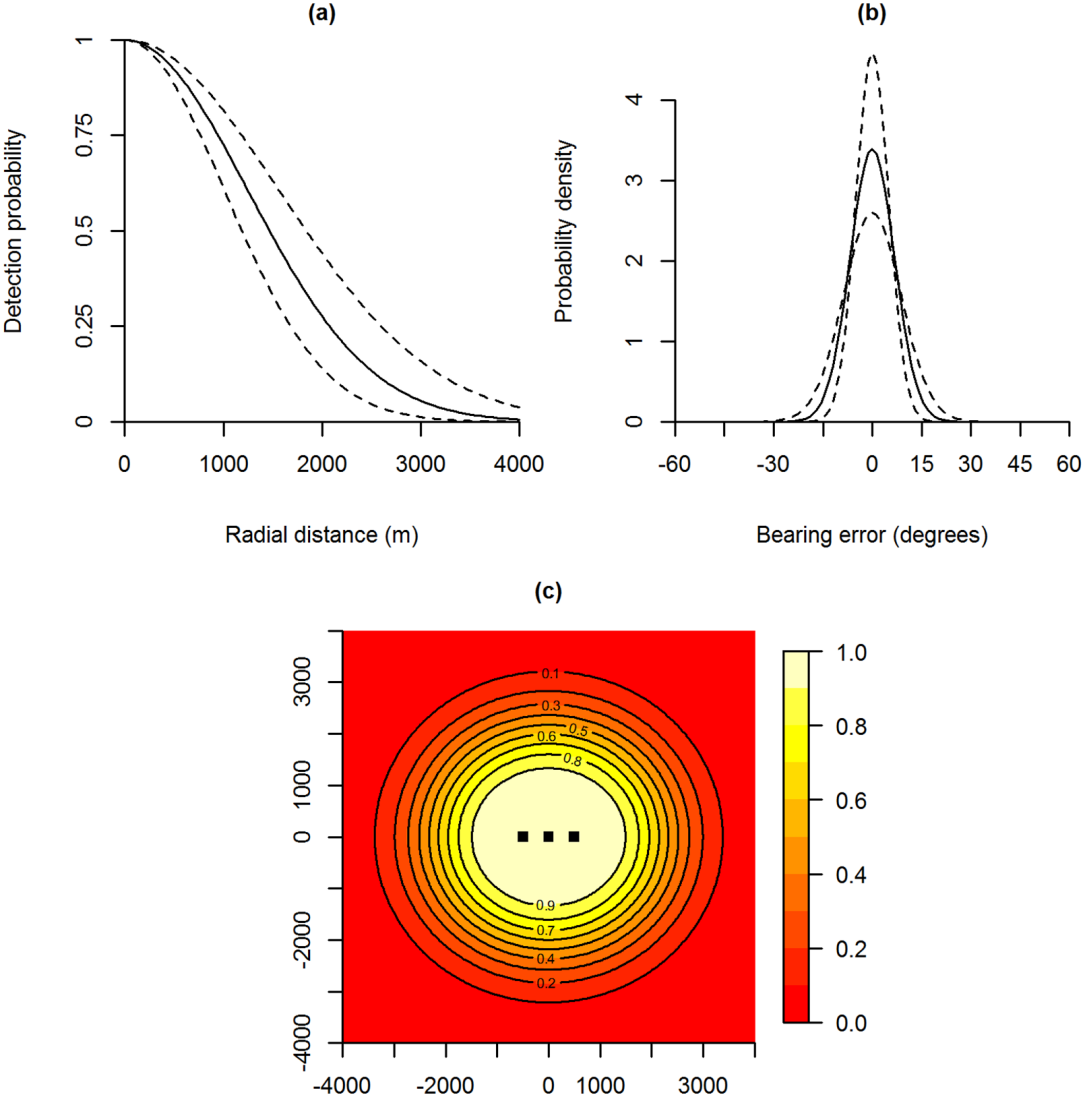
Results of the case study analysis. Fitted detection function (a), bearing error distribution (b) and detection surface for the first array (c) for the preferred model. Dotted lines in plots (a) and (b) show 95% parametric bootstrap confidence intervals. Axis units in plot (c) are in metres.

The model with the lowest AIC score had a half normal detection function and the von Mises distribution for the bearing estimates. A summary of parameter estimates for this model is given in Table 1 and Fig 2 illustrates the fitted sub-models and parametric bootstrap intervals.

**Table 1:** Results of the case study analysis. Parameter estimates and parametric bootstrap intervals for the preferred model. Density units are the number of calling groups km^−2^ and the units of the detection function scale parameter *θ*_1_ are in metres.

Parameter	Estimate	Lower 95	Upper 95
Density of calling groups (*ϕ*)	0.3197	0.1916	0.4925
Detection function scale (*θ*_1_)	1247	1009	1563
Bearing error scale (*γ*_1_)	72.44	42.66	132.60

The estimated density was approximately 0.32 calling groups km^−2^ and the detection function scale parameter was approximately 1250m, which is consistent with prior expert knowledge. The average effective sampling area for each 1 by 3 array of listening posts was therefore estimated to be 18.5 km^2^, with a 95% CI of (12.0, 30.9).

To put our density estimate into context, we also applied two alternative methods for comparison: the traditional ‘maximum listening distance’ technique, and the ‘effective detection radius’ technique of [15]. Using the traditional approach, with a maximum listening distance of 1500m, the estimated effective sampling area was calculated to be 10.05 km^2^ per array, which resulted in a density estimate of 0.59 calling groups km^−2^. Applying the approach of [15], using the fitted detection function from Fig 2 to calculate the effective radius (which was estimated to be approximately 1760m) lead to an estimated effective sampling area of 13.25 km^2^ per array and a density estimate of 0.45 calling groups km^−2^.

### Simulation 1 results: Comparing survey designs

Results from the first simulation study are summarised in Table 2. The main conclusions from this study are:

1. SECR with bearings outperformed standard SECR for all designs. Estimator variance (measured in terms of the estimated root mean squared error) and estimator bias were both consistently higher for standard SECR. Observed bias ranged from 0.9% to 2.8% for SECR with bearings and 2.9% to 76.8% for standard SECR. The distributions for density estimates for standard SECR were also considerably more diffuse.
2. Increasing the listening post spacing reduced the variance for all designs. Increasing the listening post spacing also reduced the bias for all designs when using standard SECR; however there was no discernible effect of post spacing on bias for SECR with bearings.
3. Linear arrays yielded lower variance than non-linear arrays of the same size. For example, 1 by 3 arrays showed lower variance than triangular arrays at all post spacings. Linear arrays also yielded lower bias than non-linear arrays of equivalent size for standard SECR; however the effect of array shape on bias for SECR with bearings was unclear.
4. Larger arrays yielded lower bias and variance than smaller arrays at all post spacings, with 1 by 4 arrays outperforming 1 by 3 arrays, and 2 by 2 arrays outperforming triangular arrays.

**Table 2:** Results of the first simulation study. Percentage bias and root mean squared error (in brackets) are given for density estimates.

Model	Array	0.5 km	0.75 km	1 km
SECR + Bearings	3 by 1	2.78 (0.080)	1.92 (0.065)	1.99 (0.062)
	Triangular	1.82 (0.089)	1.97 (0.074)	1.75 (0.067)
	4 by 1	0.96 (0.057)	1.12 (0.049)	0.91 (0.046)
	2 by 2	1.08 (0.067)	1.00 (0.056)	1.15 (0.050)
SECR	3 by 1	19.73 (0.273)	6.21 (0.167)	4.19 (0.121)
	Triangular	76.73 (0.523)	23.14 (0.294)	10.27 (0.204)
	4 by 1	4.42 (0.151)	3.39 (0.096)	2.89 (0.074)
	2 by 2	30.29 (0.326)	10.54 (0.199)	3.26 (0.132)

Given the performance of the alternative survey designs when using SECR with bearings, these results suggest that the design used in the survey is slightly suboptimal. Overall, the best listening post arrangement of those investigated appears to be the 1 by 4 array with a post spacing of 1000m. However, even at 500m spacing the 1 by 4 array outperformed both the 1 by 3 and triangular arrays at 1000m spacing, suggesting that increasing array size might lead to a greater improvement in performance than increasing the post spacing within each array.

### Simulation 2 results: Stochastic availability

Results from the simulation study with stochastic availability are summarised in Table 3. The main conclusions from this study are:

1. The observed bias for all model parameters was low—i.e. less than 1%.
2. Estimation of the detection function intercept *θ*_0_ was not confounded with the availability parameter *ρ*—i.e. there were no issues with parameter identifiability.

**Table 3:** Results of the second simulation study. Bias, root mean squared error (RMSE) and coefficient of variation (CV) are shown for all model parameters. Each simulation used three sampling occasions and 13 replicates of a 1 by 4 array, with 1000m spacing between listening posts in each array.

Parameter	Bias (%)	RMSE	CV (%)
Density of groups (*ϕ*)	0.93	0.08	11.94
Detection function intercept (*θ*_0_)	0.16	0.05	6.88
Detection function scale (*θ*_1_)	0.05	0.06	4.63
Bearings scale (*γ*)	-0.26	9.64	13.41
Calling probability (*ρ*)	0.06	0.05	9.46

## Discussion

### Case study analysis

Our density estimate of 0.32 calling groups km^−2^ was considerably lower, and our estimate of 18.5 km^2^ for the effective sampling area considerably higher, than that of both of the alternative techniques which were applied for comparison. The SECR results therefore imply that, at least for this study population and the survey design used, the maximum listening distance and effective listening radius techniques may both be prone to positive bias due to underestimation of the effective sampling area. The degree of bias in this case was most severe for the maximum listening distance method, although in general we would expect the size and direction of bias associated with this technique to depend on the true detection function and the number and spacing of listening posts within each array. For the the effective listening radius method we would expect the degree of bias to be worse for larger arrays, smaller listening post spacings, non-linear arrays and wider detection functions. There is also likely to be an additional source of error associated with the estimated distances which are required by the effective radius method in order to fit a detection function (in our comparison we used the estimated detection function obtained from the SECR analysis).

Drawing comparisons between our density estimate and the results of previous surveys of *Nomascus* gibbons is problematic as the genus has suffered significant population declines across its range with population densities being largely determined by local threat levels [28]. However the Veun Sai-Siem Pang Conservation Area, where this study was conducted, is considered to have close to natural densities with little evidence of recent historical hunting. In this case the most relevant population for comparison, due to its broadly similar habitat, low threat levels and close phylogenetic relationship to *N. annamensis*, is that of *N. gabriellae* in Seima Protection Forest, Mondulkiri Province, Cambodia. [18] estimated the density of this population to be approximately 0.40 calling groups km^−2^, using 24 single-post arrays and a listening distance of 1500m, which is broadly consistent with our estimate of 0.32 calling groups km^−2^. However, without information on the shape of the detection function, the degree and direction of possible bias for the *N. gabriellae* estimate is difficult to determine.

### Simulation study to compare survey designs

The results of the first simulation study suggest that the survey design used in the case study may lead to a 2–3% positive bias when estimating calling group density using SECR with bearings. Our results suggest that relatively modest alterations to this design are likely to improve precision and reduce bias. However, whether or not these alterations are worth the efficiency gains in practice will depend on the additional costs involved, e.g. in terms of staff resources and transit time required to reach the listening posts.

In general, the optimal spacing of detectors is likely to depend on the detection function scale parameter. Detectors need to be far enough apart that detection probability falls off very substantially over their range (otherwise there is insufficient information in detections about the detection function form). They also need to be close enough together to generate a sufficient number of recaptures on different detectors. The optimal arrangement of listening posts may therefore depend on factors such as the vocalisation propagation distance of the study species and the characteristics of the habitat being surveyed; for example, if the range of the detection function is relatively narrow then closer spacings may be preferred. However, for a detection function scale parameter of 1250m, our results suggest that increasing the listening posts spacing from 500m to either 750m or 1000m would be likely to improve estimator precision.

Sub-optimal listening post spacing also provides a possible explanation for why the linear arrays tended to outperform non-linear arrays of the same size; since linear arrays had larger maximum distances between posts they would therefore have contained more information on the form of the detection function.

### Simulation study with stochastic availability

In addition to choosing an appropriate array design we also recommend the use of the multi-occasion SECR model with stochastic availability for future population assessment of gibbon species. Providing groups can be identified across days, this approach allows estimation of group density via simultaneous estimation of the proportion of groups calling on a given day. This is a better approach than estimating calling probability externally to the acoustic survey, since individual, temporal and spatial variation may render such estimates unsuitable.

The simulation with stochastic availability also shows that the detection function intercept, *θ*_0_, can be estimated at the same time as calling probability. The ability to estimate *θ*_0_ is likely to be useful for multi-occasion surveys of gibbons. For a multi-occasion SECR model we assume that groups have a fixed home range centre, as opposed to fixed physical locations. This distinction is important for gibbon groups since they are unlikely to occupy the same physical location within their home ranges on each occasion. For a multi-occasion survey the detection function is therefore a combination of the probability of detecting a group, given a group’s physical location, and the movement of groups between occasions. In this case *θ*_0_ represents the detection probability of a calling group whose *home range centre* is at zero distance from the listening post, and is unlikely to equal 1.

### Extensions and applications

A useful extension to the approach outlined here would be to include covariates (although these were not available for our data). For example, heterogeneity in calling probability could be incorporated via a inverse logit transform of a linear combination of covariates such as site, season and weather conditions. Similar modelling approaches could be used to estimate detection function and bearing error scale parameters separately for each observer in order to account for differences in expertise. Estimated distances could also be incorporated in addition to estimated bearings—for example using a gamma or log-normal distribution—since these data can quite easily be collected in the field. However, the potential improvement this additional information might confer in terms of the precision of the density estimate will depend on the precision of the estimated distances.

Remote acoustic recording devices, which have been used in previous population assessments of primates [29] and SECR studies of non-primate species (e.g. [7, 9]), may be a potential alternative method of data collection that could eliminate the effects of any observer bias. Provided recaptures can be accurately identified, supplementary data on group location such as signal strength and time-of-arrival could then be used within the modified SECR framework in place of estimated bearings and distances.

The chance of capture from acoustic surveys is generally high for gibbons, given a sufficiently long observation period within each sampling occasion and provided that the survey avoids rainy periods and is not in areas with high hunting pressure (both of which will suppress vocalisation frequency). However, in cases where small sample size is due to low recapture rates this may be compensated by optimising the survey design (e.g. using closer listening post spacings) and in cases where the underlying population density is low this may require the use of additional listening posts per array or additional arrays. Whilst it is not standard practice for surveys of gibbon species, the use of playbacks to induce vocalisations may also be an option worth considering in cases when the survey design cannot be optimised to yield an adequate sample size.

We also note that the methods outlined above provide estimates of density for the covered area only. Extrapolation to the wider survey area would need to account for the additional component of variance due to inter-array variation in the encounter rate. However, methods for incorporating this additional component of variance will depend on the survey design (e.g. in terms of the number and placement of the arrays) are yet to be developed in the context of SECR models.

In addition to gibbons, population assessments for a variety of vocally territorial primate species have also been conducted by mapping estimated locations via triangulation of calls, with examples including Dian’s tarsier *Tarsius dianae* [30], indri *Indri indri* [31], black howler monkey *Alouatta pigra* [32] and Andean titi monkey *Callicebus oenanthe* [33]. Similar survey techniques to those described here have also been used for vocalising non-primate species such as coyote *Canis latrans* [34]. We anticipate that the SECR methods outlined above will provide a viable means of obtaining reliable density estimates for such species.

## Supporting Information

S1 Appendix Supplementary details on the case study analysis. This includes listening post UTM coordinates, starting values used for the fitting procedure, the rationale for the choice of integration grid and AIC values for all candidate models.
(PDF)

S2 Appendix Derivation of components for SECR likelihood with stochastic availability. Derivation of Eqs 12 and 14.
(PDF)
