# Supplementary material for: An Efficient Acoustic Density Estimation Method with Human Detectors Applied to Gibbons in Cambodia
Source: PLoS One. 2016 May 19;11(5):e0155066. doi: 10.1371/journal.pone.0155066 (PMC4873237; doi:10.1371/journal.pone.0155066)
Supplement: S1 Appendix — This includes listening post UTM coordinates, starting values used for the fitting procedure, the rationale for the choice of integration grid and AIC values for all candidate models. (PDF) [file pone.0155066.s001.pdf]

Appendix S1: Supplementary details on the case study analysis

Table S1.1. UTM 48 coordinates of the listening posts. The number component of the Id column gives the Id of the array.

Id	UTM.x	UTM.y
2A	683869.0	1551956
2B	684369.0	1551956
2C	684869.0	1551956
3A	690078.4	1551956
3B	690580.0	1551986
3C	691078.0	1551956
4A	677660.0	1557165
4B	678176.0	1557202
4C	678660.0	1557165
5A	683848.0	1557157
5B	684336.0	1557183
5C	684885.0	1557167
6A	690085.0	1557174
6B	690570.0	1557163
6C	691082.0	1557128
7A	696287.0	1557165
7B	696787.0	1557165
7C	697287.0	1557165
10A	683851.0	1562357
10B	684360.0	1562363
10C	684837.0	1562394
11A	690084.0	1562380
11B	690578.0	1562383
11C	691078.0	1562369
12A	696287.4	1562374
12B	696787.4	1562374
12C	697287.0	1562374
13A	665242.0	1567583
13B	665742.0	1567583
13C	666242.0	1567583
15A	677658.0	1567594
15B	678153.0	1567586
15C	678663.0	1567582
16A	683890.0	1567588
16B	684367.0	1567582
16C	684875.0	1567586
17A	690090.0	1567557
17B	690586.0	1567576
17C	691061.0	1567592

Starting values

The nlm optimization algorithm required the selection of starting values for all estimated parameters. Starting values for the detection function were chosen to reflect prior knowledge of the observation process gained in the field, such that the majority of calling groups with radial distances in the range of 0-1000m would be detected and that detection probability for calling groups at or beyond 3000m was virtually zero. The starting value for the density parameter, ϕ, was set to 0.5 km^{-2} and the scale parameters for the bearing error distributions were chosen such that the majority of values fell within within approximately $\pm$ 60 degrees from zero. Fig S1.1 provides an illustration of the detection and bearing error starting values and Table S1.2 gives a list of starting values for all candidate models.

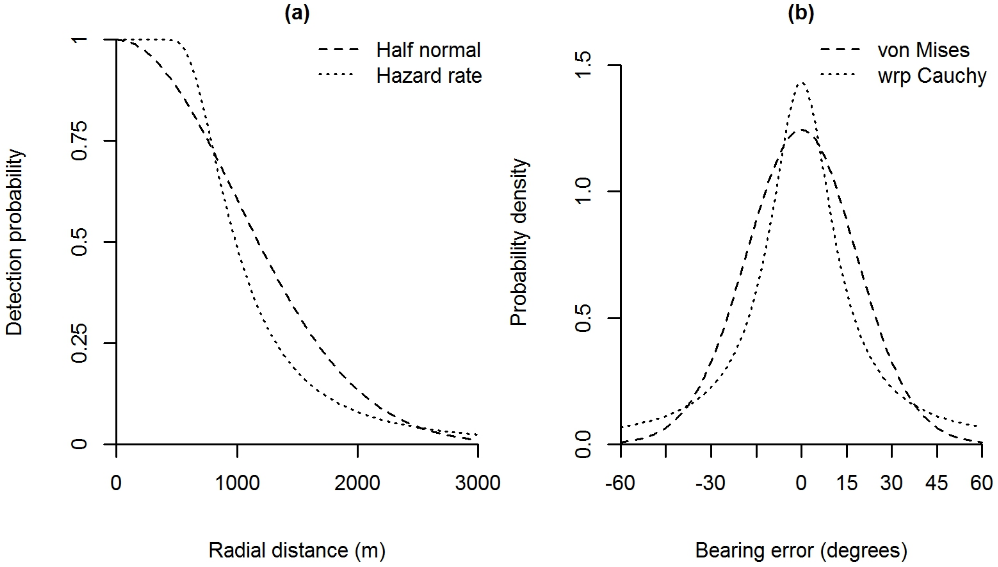

Figure S1.1. Starting values for candidate model parameters. Plot (a) shows detection function candidate models, plot (b) shows bearing error candidate models.

Table S1.2. Starting values for candidate model parameters. Density units are the number of calling groups km^{-2} and the units of the detection function scale parameter θ_1 are in metres. $E[n]$ gives the expected number of detected groups.

**Detection function**	**Bearings model**	ϕ	θ_1	θ_2	γ	$E[n]$
Half normal	von Mises	0.5	1000	-	10.0	78.8
Half normal	wrapped Cauchy	0.5	1000	-	0.8	78.8
Hazard rate	von Mises	0.5	875	3.00	10.0	78.4
Hazard rate	wrapped Cauchy	0.5	875	3.00	0.8	78.4

Choice of integration grid

Numerical integration was carried out using a grid of points, referred to here as a ‘mask’. The mask defines a region within which the unobserved locations are assumed to exist and is specified using a buffer distance around the array and the distance between consecutive grid points. Separate masks were constructed for each array.

Since estimates and computation time may be sensitive to the choice of mask the performance of a selection of mask designs was assessed. Fig S1.2 shows estimates obtained from fitting one of the candidate models using a series of different mask sizes and resolutions. Estimates appear to stabilise for spacings of 100m or less and buffer sizes of 5000m and above appear to be sufficiently large to avoid introducing bias. For the case study analysis we chose a buffer distance of 6000m and a mask point spacing of 50m, which resulting in a grid of approximately 50000 points per array.

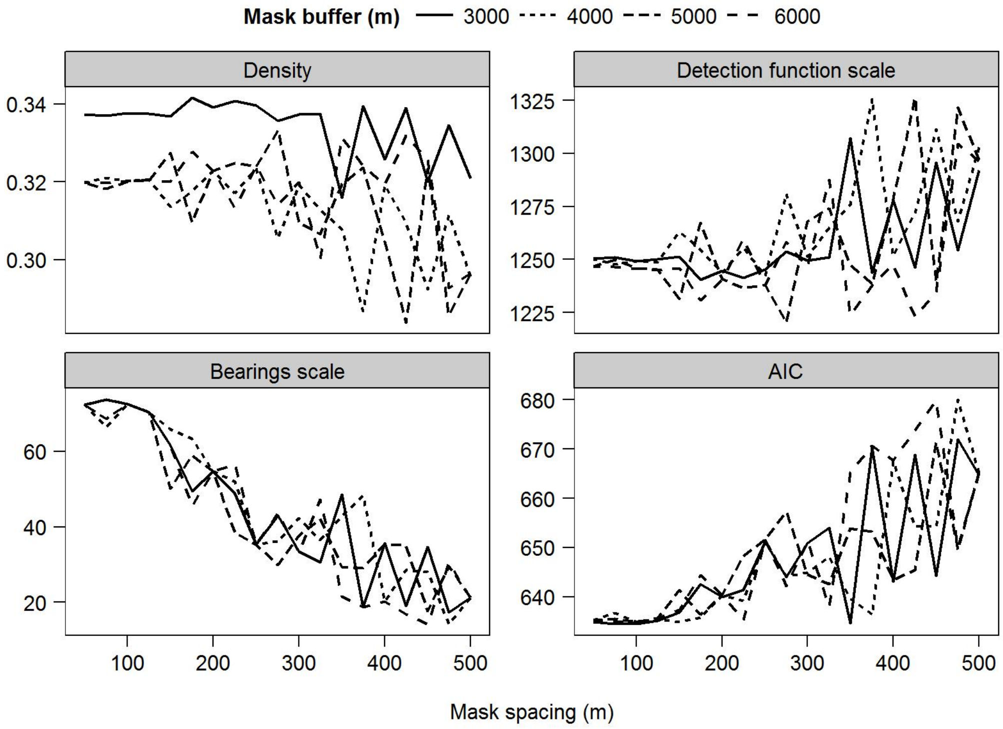

Figure S1.2. Effect of integration grid on parameter estimates. Estimates and model AIC values obtained from model fitting using the half normal detection function and the von Mises bearings distribution for a variety of integration grids.

Using this technique for numerical integration, the actual likelihood used in the analysis was an approximation to Eq (1), with summation over the mask points used to approximate the integration step. The actual likelihood used converges to the likelihood in Eq (1) as the buffer tends to infinity and the mask point spacing tends to 0. This technique was also used by [?] who presented an explicit form of the approximated likelihood.

Candidate models

Table S1.3 shows the full set of parameter estimates for the candidate models fitted in the case study analysis.

Table S1.3. Parameter estimates for candidate models. Density units are the number of calling groups km^{-2} and the units of the detection function scale parameter, θ_1, are in meters.

**Detection function**	**Bearings model**	ϕ	θ_1	θ_2	γ	ΔAIC
Half normal	von Mises	0.32	1246.58	-	72.44	0
Hazard rate	von Mises	0.33	1281.43	3.60	50.16	10.6
Half normal	wrapped Cauchy	0.28	1338.01	-	0.96	12.5
Hazard rate	wrapped Cauchy	0.27	1331.02	3.33	0.95	24.6

References

[1] Efford MG, Dawson DK, Borchers DL. Population density estimated from locations of individuals on a passive detector array. Ecology. 2009 Oct;90(10):2676–2682.
