# Supplementary material for: An Efficient Acoustic Density Estimation Method with Human Detectors Applied to Gibbons in Cambodia
Source: PLoS One. 2016 May 19;11(5):e0155066. doi: 10.1371/journal.pone.0155066 (PMC4873237; doi:10.1371/journal.pone.0155066)
Supplement: S2 Appendix — Derivation of Eqs 12 and 14. (PDF) [file pone.0155066.s002.pdf]

Appendix S2: Derivation of components for SECR likelihood with stochastic availability

Derivation of Eq (12)

We define the indicator variable $\omega_{i.s}$ which takes the value 1 if animal i was detected on occasion s and 0 otherwise. The detection surface can be expressed in terms of this indicator variable as,

$$p.(\boldsymbol{x}_i; \boldsymbol{\theta}, \rho) = 1 - \prod_{s=1}^{S} P(\omega_{i.s} = 0 \mid \boldsymbol{x}_i, \boldsymbol{\theta}, \rho),$$

which gives the probability that animal i was detected by at least one detector on at least one occasion.

To derive an expression for $P(\omega_{i.s} = 0 \mid \boldsymbol{x}_i, \boldsymbol{\theta}, \rho)$ we need to define the joint probability of $\omega_{i.s} = 0$ and the random effect α_{is} (which takes value 1 if animal i was available on occasion s and 0 otherwise) and then sum over all possible values of α_{is}. Omitting the parameters $\boldsymbol{x}_i$, $\boldsymbol{\theta}$ and ρ for brevity we have,

$$\begin{aligned}
P(\omega_{i.s} = 0) &= \sum_{\alpha_{is}=0}^{1} P(\alpha_{is}, \omega_{i.s} = 0) \\
&= \sum_{\alpha_{is}=0}^{1} P(\alpha_{is}) P(\omega_{i.s} = 0 \mid \alpha_{is}) \\
&= P(\alpha_{is} = 0) P(\omega_{i.s} = 0 \mid \alpha_{is} = 0) + P(\alpha_{is} = 1) P(\omega_{i.s} = 0 \mid \alpha_{is} = 1).
\end{aligned}$$

Recalling that $\rho = P(\alpha_{is} = 1)$, and recognising that $P(\omega_{i.s} = 0 \mid \alpha_{is} = 0) = 1$, since an animal cannot be detected if it doesn't call, this expression simplifies to,

$$P(\omega_{i.s} = 0) = (1 - \rho) + \rho P(\omega_{i.s} = 0, \alpha_{is} = 1).$$

We can also re-write $P(\omega_{i.s} = 0, \alpha_{is} = 1)$, the probability that animal i was not detected at any detector on occasion s (given that it was available on that occasion), in terms of the detection function,

$$P(\omega_{i.s} = 0, \alpha_{is} = 1) = \prod_{k=1}^{K} [1 - p_{ks}(\boldsymbol{x}_i, \boldsymbol{\theta} \mid \alpha_{is} = 1)].$$

Combining the above components we derive the full expression,

$$p.(\boldsymbol{x}_i; \boldsymbol{\theta}, \rho) = 1 - \prod_{s=1}^{S} \left\{ (1 - \rho) + \rho \prod_{k=1}^{K} [1 - p_{ks}(\boldsymbol{x}_i; \boldsymbol{\theta} \mid \alpha_{is} = 1)] \right\}.$$

Derivation of Eq (14)

We define the indicator variable δ_i which takes the value 1 if animal i was detected during the survey and 0 otherwise. Using Bayes' theorem, the conditional probability for the capture history for animal i can be expressed using this indicator as,

$$P(\boldsymbol{\omega}_i \mid \delta_i = 1) = \frac{P(\delta_i = 1 \mid \boldsymbol{\omega}_i) P(\boldsymbol{\omega}_i)}{P(\delta_i = 1)},$$

omitting the parameters $\boldsymbol{x}_i$, $\boldsymbol{\theta}$ and ρ for brevity. It follows that $P(\delta_i = 1 \mid \boldsymbol{\omega}_i) = 1$, since animal i must have been detected at least once given that it has a non-zero capture history for the entire survey. We already have an expression for $P(\delta_i = 1)$, given location $\boldsymbol{x}_i$, in the form of the detection surface, $p.(\boldsymbol{x}_i; \boldsymbol{\theta}, \rho)$. The unconditional probability of $\boldsymbol{\omega}_i$ can also be re-expressed as the product of the unconditional probabilities of the capture histories for each occasion (assuming independence),

$$P(\boldsymbol{\omega}_i \mid \delta_i = 1) = \frac{\prod_{s=1}^{S} P(\boldsymbol{\omega}_{is})}{p.(\boldsymbol{x}_i; \boldsymbol{\theta}, \rho)}.$$

To derive an expression for $P(\boldsymbol{\omega}_{is})$ we need to express the joint probability of $\boldsymbol{\omega}_{is}$ and the random effect α_{is} (which takes value 1 if animal i was available on occasion s and 0 otherwise) and then sum over all possible values of α_{is}.

$$\begin{aligned} P(\boldsymbol{\omega}_{is}) &= \sum_{\alpha_{is}=0}^{1} P(\alpha_{is}, \boldsymbol{\omega}_{is}) \\ &= \sum_{\alpha_{is}=0}^{1} P(\alpha_{is}) P(\boldsymbol{\omega}_{is} \mid \alpha_{is}) \\ &= P(\alpha_{is} = 0) P(\boldsymbol{\omega}_{is} \mid \alpha_{is} = 0) + P(\alpha_{is} = 1) P(\boldsymbol{\omega}_{is} \mid \alpha_{is} = 1) \end{aligned}$$

Recalling that $\rho = P(\alpha_{is} = 1)$, this simplifies to,

$$P(\boldsymbol{\omega}_{is}) = (1 - \rho) P(\boldsymbol{\omega}_{is} \mid \alpha_{is} = 0) + \rho P(\boldsymbol{\omega}_{is} \mid \alpha_{is} = 1)$$

The value of $P(\boldsymbol{\omega}_{is} \mid \alpha_{is} = 0)$, which gives the probability of the capture history given that the animal did not call, will simplify to either 1 or 0 depending on whether or not the animal was detected. If the animal was not detected then $\boldsymbol{\omega}_{is}$ will be zero for all detectors and $P(\boldsymbol{\omega}_{is} \mid \alpha_{is} = 0)$ will be equal to 1. However, if the animal was detected then $P(\boldsymbol{\omega}_{is} \mid \alpha_{is} = 0)$ will be 0 (since a non-zero capture history is impossible if the animal did not call). These two possibilities can be described in terms of the indicator variable $\omega_{i.s}$ (which takes value 1 if animal i was detected on occasion s and 0 otherwise),

$$\begin{aligned} P(\boldsymbol{\omega}_{is} \mid \omega_{i.s} = 0) &= (1 - \rho) + \rho P(\boldsymbol{\omega}_{is} \mid \alpha_{is} = 1) \\ P(\boldsymbol{\omega}_{is} \mid \omega_{i.s} = 1) &= \rho P(\boldsymbol{\omega}_{is} \mid \alpha_{is} = 1) \end{aligned}$$

We can therefore use the observed value of $\omega_{i.s}$ to switch on or off the $(1 - \rho)$ term,

$$P(\boldsymbol{\omega}_{is}) = (1 - \rho)(1 - \omega_{i.s}) + \rho P(\boldsymbol{\omega}_{is} \mid \alpha_{is} = 1).$$

Finally, we obtain an expression for $P(\boldsymbol{\omega}_{is} \mid \alpha_{is} = 1)$ using the detection function (and assuming independent detections),

$$P(\boldsymbol{\omega}_{is} \mid \alpha_{is} = 1) = \prod_{k=1}^{K} Bern(\omega_{iks} \mid p_{ks}(\boldsymbol{x}_i; \boldsymbol{\theta} \mid \alpha_{is} = 1))$$

Combining the above components we derive the full expression,

$$P(\boldsymbol{\omega}_i \mid \boldsymbol{x}_i; \boldsymbol{\theta}, \rho) = \frac{\prod_{s=1}^{S} \left\{(1-\rho)(1-\omega_{i.s}) + \rho \prod_{k=1}^{K} Bern(\omega_{iks}, p_{ks}(\boldsymbol{x}_i; \boldsymbol{\theta} \mid \alpha_{is} = 1))\right\}}{p.(\boldsymbol{x}_i; \boldsymbol{\theta}, \rho)}$$
